# Quantum scattering of icosahedron fullerene C_60_ with noble-gas atoms

**DOI:** 10.1038/s41598-024-59481-x

**Published:** 2024-04-23

**Authors:** Jacek Kłos, Eite Tiesinga, Svetlana Kotochigova

**Affiliations:** 1 Department of Physics, Temple University, Philadelphia, PA 19122 USA; 2 Joint Quantum Institute, College Park, MD 20742 USA; 3 National Institute of Standards and Technology, Gaithersburg, MD 20899 USA

**Keywords:** Chemical physics, Atomic and molecular collision processes, Quantum chemistry

## Abstract

There exist multiple ways to cool neutral molecules. A front runner is the technique of buffer gas cooling, where momentum-changing collisions with abundant cold noble-gas atoms cool the molecules. This approach can, in principle, produce the most diverse samples of cold molecules. We present quantum mechanical and semiclassical calculations of the elastic scattering differential cross sections and rate coefficients of the C_60_ fullerene with He and Ar noble-gas atoms in order to quantify the effectiveness of buffer gas cooling for this molecule. We also develop new three-dimensional potential energy surfaces for this purpose using dispersion-corrected density functional theory (DFT) with counterpoise correction. The icosahedral anisotropy of the molecular system is reproduced by expanding the potential in terms of symmetry-allowed spherical harmonics. Long-range dispersion coefficients have been computed from frequency dependent polarizabilities of C_60_ and the noble-gas atoms. We find that the potential of the fullerene with He is about five times shallower than that with Ar. Anisotropic corrections are very weak for both systems and omitted in the quantum scattering calculations giving us a nearly quantitative estimate of elastic scattering observables. Finally, we have computed differential cross sections at the collision energies used in experiments by Han et al. (Chem Phys Lett 235:211, 1995), corrected for the sensitivity of their apparatus, and we find satisfactory agreement for C_60_ scattering with Ar.

## Introduction

The discovery of the fullerene, C_60_ molecule opened up new horizons in physical and chemical research^1,2^. It has a fascinating structure due to its highest-allowed point-group symmetry, the icosahedral symmetry. Pure $^{12}$C_60_ represents a large stable molecule with no nuclear spin. These molecules can be easily formed in the gas phase^3^ as well as into a crystal^4^.

The carbon atoms in C_60_ form a cage structure of 12 pentagons and 20 hexagons. In fact, there exists a large family of fullerenes with structural variations^5,6^ of their dome shape of 12 pentagons and an increasing number of hexagons. The nearly spherical fullerene molecules have high structural and chemical stability. For example, due to their large electron affinity, these molecules can form various derivative synthetic systems, such as hetero, *endohedral fullerenes* that encapsulate a single ion, atom, or even small molecules such as H$_2$O or HF^7,8^ or larger ones such as Sc$_3$N^9^. Recently endohedral formaldehyde H$_2$CO@C_60_ has been synthesized ^10^ by molecular surgery ^11^. These encapsulated atoms or molecules maintain many of original properties and are isolated from the wider environment. Endohedral fullerenes have stimulated research in the field of fullerene-based nanotechnology^12–16^.

A second class of synthetic systems are *exohedral fullerenes*, where ligand atoms or molecules are attached to the outside of the fullerene shell^17–19^. These exohedral systems can posses strong interactions and bonding changing the nature of the ligands. The dominant reason for these changes is that the electron wavefunctions of the hybridized carbon atoms in fullerenes have a $sp^{3}$ character with the spatial lobes of the *p*-wave electron orbitals more outside than inside the “sphere”^16^.

For the design of novel endohedral fullerenes a deep understanding of the targeted electronic structure as well as transport and optical properties is required. Exploiting the unique ability of fullerenes to stabilize internal metallic molecules is essential for the control and manipulation of their electronic and spin states ^20^. Currently, the primary goal of endohedral and exohedral fullerene studies is to put forward a fully quantitative, quantum description of these systems.

Gas phase fullerenes have also inspired experimental interests. Their ro-vibrational structure was elucidated in Refs.^21,22^. Recently, single quantum state preparation and control was achieved using high-resolution spectroscopy of the gas-phase C_60_ molecules^23,24^. These experiments showed an unusual spectrum of rotational transitions associated with the symmetry-induced restrictions on the fullerene motion. Finally, fullerene spectroscopy has also made a noticeable impact on astrophysicists studying the infrared emission from large carbon clusters in the intergalactic medium^25,26^. Additionally, the presence of the C$_{60}^+$ cation in diffuse interstellar clouds has been confirmed in Ref.^27^ by a comparison to observed bands in photofragmentation of C$_{60}^+$He in laboratory experiments by Ref.^28^.

In this paper, we investigate theoretical aspects of efficient buffer-gas or sympathetic cooling of the translational motion of neutral fullerene molecules with noble-gas atoms as the buffer gas. Buffer gas cooling of alkali-metal atoms and a “simple” diatomic molecule was first developed and demonstrated in 2005^29^. We focus on elastic and thus momentum-changing collisions of C_60_ with $^4$He and $^{40}$Ar atoms temporarily creating $^{12}$C_60_-$^4$He and $^{12}$C_60_-$^{40}$Ar complexes. In these collisions the noble-gas atom accepts kinetic energy from the molecule and thus leaves a colder molecule behind.

We first perform quantum-chemical, electronic-structure simulations of $^{12}$C_60_-$^4$He and $^{12}$C_60_-$^{40}$Ar complexes using Density Functional Theory (DFT)^30^. Using the resulting anisotropic ground-state electronic potential energy surface, we then apply a numerical quantum-mechanical scattering solver to determine collisional properties of these molecular systems. Our results on the small inelastic rate coefficients have already been described in Ref.^24^. For these calculations, we assume that the temperature of C_60_ is well below that of room temperature following, for example, the 150 K experimental conditions in Ref.^23^. At these temperatures we can concentrate on the thermal population of the rotational states of the energetically-lowest “$v=0$” vibrational state of C_60_. We find that the collision cross section and rate coefficient are dominated by elastic scattering from the isotropic component of the interaction potentials. Relative strengths of isotropic and anisotropic components of the interaction potentials can be found in our recent publication^24^. Here, we compare the quantum results to those from a much simpler semi-classical scattering model, which assumes that the scattering between C_60_ and a noble-gas atom is dominated by isotropic long-range dispersion forces.

We also comment on the difference of our elastic rate coefficients for C_60_ and noble-gas atoms with those from a previous theoretical study^31^ based on pair-wise Lennard-Jones potentials. Finally, we compare our data with the experimental molecular-beam observations by Han et al.^32^ for C_60_-Ar. They measured apparent, detector-limited elastic cross sections at four high collision energies. The agreement between our theory and this experiment is satisfactory. We note that there exist few other studies on scattering processes involving the neutral fullerene molecule. We can mention molecular dynamics calculations at high collision energies between C_60_ and noble gases in Ref.^33^ as well as molecular dynamics simulations for C_60_ and nitrogen atoms (See Ref.^34^ and references therein).

## Results and discussions

### Potential energy surfaces of C_60_ and an Ar or He atom

**Figure 1:**
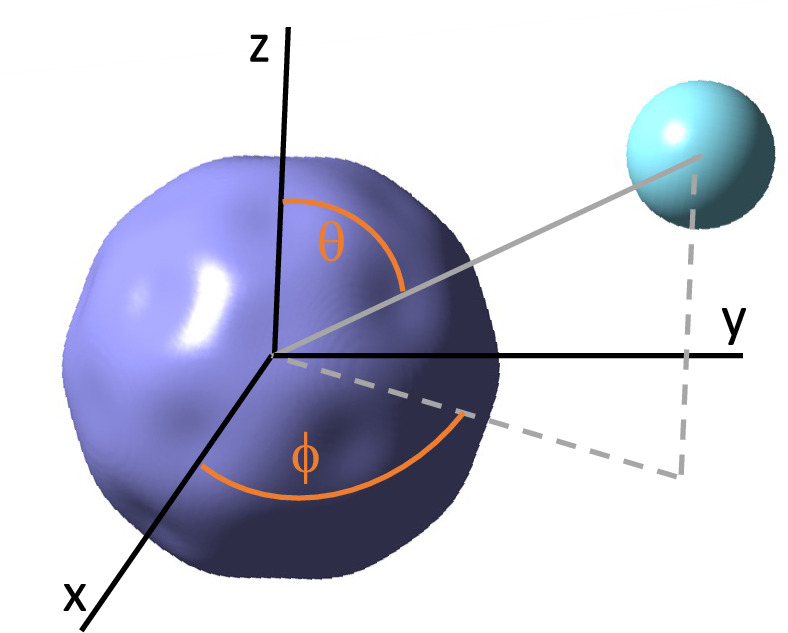
Cartesian ${{\textbf{x}}=(x,y,z)}$ and spherical $(R,\theta ,\phi )$ coordinate systems for the noble-gas atom (cyan sphere) near C_60_ used in our DFT simulations. The origin of the coordinate systems corresponds to the center of mass of the fullerene and ${R=|\textbf{x}|}$ is the separation between the noble-gas atom and the center of mass of C_60_. The *x* and *z* axes coincide with a two-fold and five-fold symmetry axis of the fullerene, respectively. The figure also shows an isosurface (purple) of the electronic potential $U(\textbf{x})$ when a noble-gas atom goes “around” the fullerene at a fixed separation *R* close to the equilibrium separation.

For the relevant collision energies and in anticipation of the fact that bonding with noble-gas atoms tends to be weak, we assume throughout this paper that the carbon atoms in C_60_ are frozen at their equilibrium geometry and satisfy icosahedral symmetry. A weak van-der-Waals bond implies negligible displacements of carbon atoms even at the closest approach of the noble-gas atom. Convenient three-dimensional Cartesian and spherical coordinate systems, ${{\textbf{x}}=(x,y,z)}$ and ${{\textbf{x}}=(R,\theta ,\phi )}$, respectively, locating the noble-gas atom relative to the center of mass of this “frozen” C_60_ molecule are defined in Fig. 1. Electronic potential energy surfaces $U({\textbf{x}})$ are then only functions of these coordinates.

**Figure 2:**
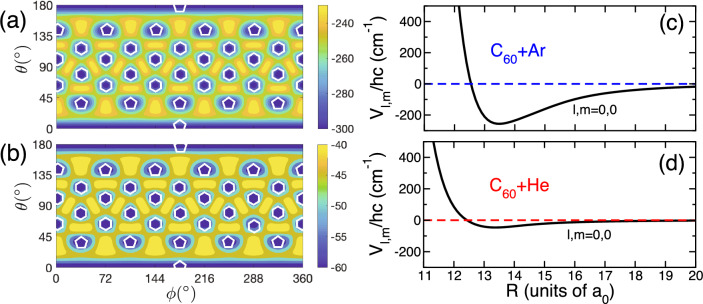
Potential energy surfaces for C_60_-Ar and C_60_-He. Panels (**a**) and (**b**) show contour plots of the potentials as functions of angles $\theta$ and $\phi$ for C_60_-Ar and C_60_-He, respectively. The separations between Ar and C_60_ and between He and C_60_ are their equilibrium bond lengths. A small white regular pentagon or hexagon is placed at each (local) minimum of the potential. They indicate that the minimum occurs when the noble-gas atom is placed above the center of one of these figures. Notice the very different energy scales of the two panels. The zero of energy occurs where the noble-gas atom is infinitely far away from the fullerene. Panels (**c**) and (**d**) show radial strengths $V_{0,0}(R)$ as functions of *R* for C_60_-Ar and C_60_-He, respectively. This isotropic strength dominates in the atom-fullerene bond. Here, $a_0=0.05291\dots$ nm is the Bohr radius.

Figure 2a and b show cuts through the electronic ground-state potential energy surface in spherical coordinates at the equilibrium separation of Ar and He with respect to the center of mass of C_60_, respectively. For C_60_-Ar and C_60_-He, these radii correspond to $R_\mathrm{eq}=7.2$ Å and 7.0 Å, respectively, where 1 Å or 1 Angstrom is $10^{-10}$ m. These equilibrium separations are about twice the average geometric radius of the fullerene. In addition, we show an isosurface, a surface of constant potential energy, for C_60_-Ar near its equilibrium separation in Cartesian coordinates in Fig. 1. We have obtained these cuts and, in fact, the full three-dimensional potentials using complete counterpoise corrected DFT calculations using the Gaussian-09 program^35^ (Any mention of commercial products is for information only; it does not imply recommendation or endorsement by the National Institute of Standards and Technology.) employing the hybrid wB97XD functional and 6-31G(d,p) basis set. Technical particulars about the DFT calculations are given in Supplemental Materials.

The isosurface in Fig. 1 has “dimples” corresponding to locations along which the noble-gas atom can approach the fullerene closer than for other geometries. In fact, the most pronounced dimples occur at the center of each hexagonal face of C_60_. Slightly weaker dimples occur at the center of each pentagonal face. The highly-symmetric form of the potential energy surfaces is better observed in Fig. 2a and b. The blue colored regions in both panels correspond to absolute or local minima of the potential. Local maxima occur in between the minima. The potential depth at the maxima relative to that at the dissociation limit is only 20–30% smaller than that at minima. Figure 2a and b also show that the potentials are shallow with depths $\approx hc\times 300$ cm$^{-1}$ for C_60_-Ar and $\approx hc\times 60$ cm$^{-1}$ for C_60_-He. Here, *h* is the Planck constant and *c* is the speed of light in vacuum.

For the scattering calculations it is convenient to expand the potential energy surfaces in terms of a sum of Racah-normalized spherical harmonics $C_{lm}(\theta ,\phi )$ that fulfill the condition $C_{lm}(0,0)=\delta _{m0}$. In fact, we write

1 $$\begin{aligned} U(\textbf{x})=\sum _{l,m\in \mathcal{L}} V_{l,m}(R) \frac{C_{lm}(\theta ,\phi )+C_{l{-m}}(\theta ,\phi )}{2}\,, \end{aligned}$$

where $V_{l,m}(R)$ are radial strengths or radial potentials and quantum numbers *l*, *m* are taken from the set $\mathcal L$ with $l=0,6,10,12,16,18, 20, \ldots$ and $m=5n$ with $n=0,1,2,\ldots$ and $m\le l$ for the icosahedral symmetry group. In practice, it is sufficient to expand $U(\textbf{x})$ with terms up to $l=20$ and $m = 20$.

Figures 2c and d show the dominant isotropic $V_{0,0}(R)$ strength as functions of separation *R* for C_60_-Ar and C_60_-He, respectively. The nature of collisions is often controlled by the long-range, large *R* behavior of the interaction potential. For our neutral systems, this behavior is due to the van-der-Waals dispersion potential, proportional to $1/R^6$. We have estimated the relevant isotropic $l,m=0,0$ as well as anisotropic $l,m\ne 0,0$ dispersion $C_6$ coefficients for the C_60_-Ar and C_60_-He systems by calculating the dynamic dipole polarizability of C_60_ within DFT theory, using the imaginary-frequency dependent dipole polarizabilities of Ar and He of Derevianko et al.^36^, and then determined van-der-Waals $C_6$ coefficients, using the Casimir-Polder formula^37^.

We find that the isotropic $C_6$ coefficients are $2523E_\mathrm{h}a_0^6$ and $369.6 E_\mathrm{h}a_0^6$ for C_60_-Ar and C_60_-He, respectively. The anisotropic dispersion coefficients are of the order of $10^{-4} E_\mathrm{h}a_0^6$. Here, $E_\mathrm{h}$ is the Hartree energy and $a_0$ is the Bohr radius. In 1995 Han et al.^32^ estimated a value for the isotropic $C_6$ for C_60_-Ar of $2035 E_\mathrm{h}a_0^6$, about 25 % smaller than our result. A tiny anisotropic dispersion coefficient, seven orders of magnitude smaller than the isotropic one, might suggest that rotational quenching of C_60_ in collisions with Ar or He has a small probability. As the atom and molecule approach each other, however, other non-dispersive anisotropies appear, leading for example to the $\approx 30\,\%$ differences between the (local) minima and maxima near the equilibrium separations of $\approx 7$ Å and which can induce rotational transitions. This corrugation of the potential energy surface is separation dependent. For example at a separation of $R\approx 10.5$ Å the energy difference between minima and maxima is only $\approx 1$ % for both systems. This implies a rapid decrease in the anisotropy and for moderate separations the Ar or He atoms “view” the fullerene as an isotropic ball. However, determinations of quenching rate coefficients fall outside the scope of this work.

### Quantum and semi-classical scattering calculations

In scattering models the noble-gas collision partner “sees” the whole fullerene cage and undergoes elastic collisions. To estimate elastic rate coefficients we resort to two approaches. The first approach is the widely used semiclassical approximation for isotropic elastic scattering^38^, which leads to the total elastic rate coefficient

2 $$\begin{aligned} K_\mathrm{SC}(E)= v\sigma _\mathrm{SC}(E) = \kappa \left( \frac{E}{E_6}\right) ^{3/10} \frac{\hbar \beta _6}{\mu } \end{aligned}$$

at relative collision energy $E=\mu v^2/2$ with relative velocity *v*. Here, $\sigma _\mathrm{SC}(E)$ is the total semi-classical elastic cross section, dimensionless $\kappa =6.13\cdots$, $\mu$ is the reduced mass, $\hbar$ is the reduced Planck constant, $E_6=\hbar ^2/(2\mu \beta _6^2)$ is the van-der-Waals energy, and $\beta _6=(2\mu C_6/\hbar ^2)^{1/4}$ is the van-der-Waals length. Equation (2) only depends on the isotropic van-der-Waals dispersion coefficient and is valid when ${E\gg E_6}$. For concreteness we note that system parameters $(\mu , E_6, \beta _6)$ are (37.860974 u, $k_\mathrm{B}\times 0.1226$ mK, $136.6a_0$) and (3.980475 u, $k_\mathrm{B}\times 9.396$ mK, $48.12a_0$) for $^{12}$C_60_-$^{40}$Ar and $^{12}$C_60_-$^4$He, respectively. Here, u is the unified atomic mass unit and $k_\mathrm{B}$ is the Boltzmann constant.

Our second approach is a quantum scattering calculation using only the isotropic radial potential $V_{0,0}(R)$. Then, we only need to numerically solve a single radial Schrödinger equation for each (end-over-end) partial wave $\ell =0,1,2,\ldots$. That is, we find radial scattering wavefunctions $\phi_{\ell }(R)$ satisfying

3 $$\begin{aligned} \frac{d^2\phi_{\ell }(R)}{dR^2}+\left( k^2-\frac{2\mu }{\hbar ^2}V_{0,0}(R)-\frac{\ell (\ell +1)}{R^2}\right) \phi_{\ell }(R)=0\,, \end{aligned}$$

where the relative wavevector $k=\sqrt{2\mu E/\hbar ^2}$. For each partial wave $\ell$ and collision energy *E*, the differential equation in Eq. (3) is propagated from $R=8.0 a_0$ up to $R=100 a_0$ using the log-derivative method^39^ implemented within Matlab (Any mention of commercial products is for information only; it does not imply recommendation or endorsement by the National Institute of Standards and Technology.)^40^ with $100\,000$ equally spaced steps. The radial wavefunctions near $100 a_0$ are used to construct the dimensionless scattering phase shift $\delta _\ell (E)$. Following Ref.^38^, we define the polar scattering angle $\theta _\mathrm{CM}$ of the collision in the center-of-mass (CM) frame and construct the differential cross section $\textrm{d}\sigma /\textrm{d}\Omega =|f(E,\theta _\mathrm{CM})|^2$ with dimension of length squared. Here, the scattering amplitude $f(E,\theta _\mathrm{CM})$ is described in terms of a sum over $\ell$ with summants that are functions of $\delta _\ell (E)$ and $\theta _\mathrm{CM}$. For scattering from an isotropic potential the scattering amplitude is independent of the azimuthal scattering angle in the CM frame. The optical theorem then tells us that the total elastic cross section $\sigma (E)=4\pi \textrm{Im} f(E,0)/k$ and the total elastic rate coefficient $K(E)=v\sigma (E)$.

**Figure 3:**
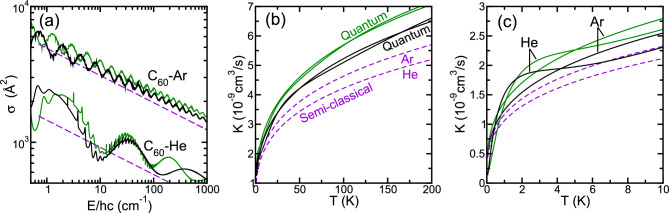
Elastic cross sections $\sigma$ as functions of relative collision energy *E* (panel **a**) and thermalized elastic rate coefficients *K* as functions of temperature *T* (panels **b** and **c**) for $^{12}$C_60_+$^4$He and $^{12}$C_60_+$^{40}\mathrm Ar$ based on quantum scattering simulations using our isotropic $V_{0,0}(R)$ strength (black solid lines) and semiclassical simulations using the corresponding isotropic van-der-Waals coefficients (purple dashed lines). The solid green lines represent our quantum simulations based on the isotropic potential derived from data in Ref.^31^. Line colors and styles are the same in all three panels. In panel (**b**) quantum-based rate coefficients for $^{12}$C_60_+$^4$He and $^{12}$C_60_+$^{40}\mathrm Ar$ are nearly indistinguishable on the scale of the figure and not further identified. In panels (**b**) and (**c**) the semiclassical predictions for He are noticeably smaller than those for Ar.

Partial waves $\ell$ up to 80 for collision energies *E* up to $hc\times 2$ cm$^{-1}$, up to 280 for *E* up to $hc\times 10$ cm$^{-1}$, and up to 2600 for *E* up to $hc\times 2000$ cm$^{-1}$ are required to converge differential-, integral cross sections, and rate coefficients for C_60_-Ar to within 1 %. To converge the C_60_-He cross sections the largest partial waves $\ell$ reach 520 at the highest collision energies. Thermally averaged quantum rate coefficients are obtained by averaging *K*(*E*) with a three-dimensional Maxwell-Boltzmann velocity distribution at temperature *T* for both fullerenes and noble-gas atoms.

### Predictions for elastic cross sections and rate coefficients

We show total elastic cross sections as functions of collision energy and thermalized rate coefficients as functions of temperature in Fig. 3a–c, respectively. For both C_60_-Ar and C_60_-He the quantum elastic cross sections in panel (a) of Fig. 3 are characterized by so-called glory oscillations^38^ in addition to narrow shape resonances due to quasi-bound states localized behind centrifugal barriers. The C_60_-He cross sections have glory oscillations with longer energy-wavelengths and larger fractional amplitudes than those for the C_60_-Ar system. The C_60_-He system also has fewer shape resonances. All these differences are consequences of the smaller reduced mass and the smaller depth of the isotropic strength $V_{0,0}(R)$ of the C_60_-He system. The semiclassical model of the cross section does not account for glory oscillations or shape resonances. It does have a $E^{-1/5}$ power-law dependence with respect to energy that qualitatively agrees with the quantum results. In fact, when we average the quantum cross section over a period of the glory oscillations, not shown in Fig. 3a, we obtain averaged cross sections as functions of energy that are $\approx 25\,\%$ larger than those from the semi-classical model.

Our thermalized elastic rate coefficient from quantum scattering and semi-classical calculations up to 200 K are shown in Fig. 3b and c. The semi-classical thermalized rate coefficient is found by performing the integration over *E* analytically. The quantum thermalized rate coefficient is determined numerically. For both systems, the glory oscillations and shape resonances have “washed out” in the averaging over the Maxwell-Boltzmann distribution. At a temperature of $T=150$ K the quantum value is around $6\times 10^{-9}$ cm$^3$s$^{-1}$ for both complexes. The semiclassical model then gives a thermalized rate coefficient of $5\times 10^{-9}$ cm$^3$s$^{-1}$ and $4\times 10^{-9}$ cm$^3$s$^{-1}$ for the C_60_-Ar and C_60_-He collisions, respectively. The larger discrepancy for C_60_-He between the quantum calculation and the semi-classical model can be associated with the fact that a smaller number of partial waves are needed to converge the cross sections than for the C_60_-Ar complex at the same collision energy or temperature. Hence, the collision of a fullerene with He is “less” classical.

Thermalization times can be estimated under the assumption that the number density of the noble-gas atoms, here $n_\mathrm{He}$ or $n_\mathrm{Ar}$, is many orders of magnitude larger that those for the fullerenes. Then the mean time between collisions for a single C_60_ molecule with noble-gas atoms at temperature *T* is $1/(K(T) n_\mathrm{He})$ or $1/(K(T) n_\mathrm{Ar})$. Taking into account that the C_60_ molecule needs to collide only a few times before the system equilibrates and using the ideal gas law, rate coefficients of a few times $10^{-9}$ cm$^3$s$^{-1}$ imply timescales of order of milliseconds at noble-gas gas pressures of order $10^{-5}$ Torr^32^. In SI units, 1 Torr is approximately 133.3 Pa.

Ruiz et al., in Ref.^31^ constructed potential energy surfaces for C_60_-He and C_60_-Ar based on the sum of sixty pair-wise Lennard-Jones potentials for C-He and C-Ar with the carbon atoms at their equilibrium position in C_60_. We expanded this potential energy surface in spherical harmonic functions and computed total elastic cross sections and thermalized rate coefficients from quantum scattering simulations using only the isotropic strength or potential $V_{0,0}(R)$. The results are also shown in Fig. 3. We observe that our predictions of cross sections and rate coefficients are smaller that those derived from data by Ruiz et al., except for temperatures below 2 K in Fig. 3c. Moreover, the glory oscillations for the two simulations in Fig. 3a are out of phase and the shape resonances occur at different collision energies.

### Comparison with a measurement in the literature

We can compare estimates of the collision-energy-dependent differential cross sections for C_60_-Ar to data obtained in supersonic-beam experiments by Han et al. ^32^. In these experiments a narrow beam of high-velocity $^{12}$C_60_ passes through a 300 K, ${\sim 10^{-5}}$ Torr pressure sample of $^{40}$Ar. Then C_60_-Ar collisions can remove C_60_ molecules from the beam and the number of remaining C_60_ molecules in the beam as function of Argon pressure is detected downstream. Han et al., kept the Ar pressure is sufficiently low that a fullerene collides at most once with the noble-gas atoms over the sample length and also realized that the Ar atoms can be considered at rest for the relevant C_60_ velocities. That is, the corresponding kinetic energies of C_60_ in their beam are much larger than those shown in Fig. 3a.

**Figure 4:**
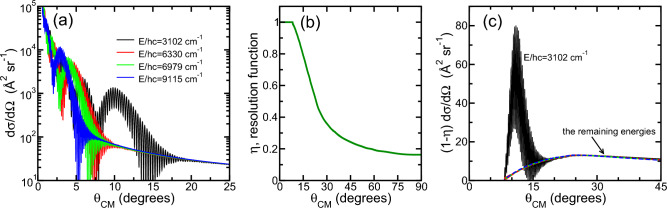
Elastic differential cross sections $\textrm{d}\sigma /\textrm{d}\Omega$ (panel **a**) and $\{1-\eta (\theta _\mathrm{CM})\} \textrm{d}\sigma /\textrm{d}\Omega$ (panel **c**) for $^{12}$C_60_-$^{40}$Ar as functions of polar scattering angle $\theta _\mathrm{CM}$ at four collision energies. The data for $\textrm{d}\sigma /\textrm{d}\Omega$ are based on quantum scattering calculations using the isotropic $V_{0,0}(R)$ potential strength. The four collision energies in the two panels correspond to the relative collision velocities of 1400 m/s, 2000 m/s, 2100 m/s, and 2400 m/s used in the supersonic-beam experiments performed by Han et al. ^32^. Panel (**b**) shows the angular resolution function $\eta (\theta _\mathrm{CM})$ of the experimental apparatus and are used in our simulations of apparent cross sections of $^{12}$C_60_-$^{40}$Ar collisions. Thus, panel (textbfc) accounts or corrects for the missing forward and small $\theta _\mathrm{CM}$ scattering that is undetectable in the experiments by Han et al. ^32^.

Figure 4a shows our differential cross section $\textrm{d}\sigma /\textrm{d}\Omega$ as functions of polar angle $\theta _\mathrm{CM}$ in the center-of-mass coordinate frame at four collision energies. These four collision energies correspond to the four relative velocities of 1400 m s^−1^, 2000 m s^−1^, 2100 m s^−1^, and 2400 m s^−1^ used by Han et al.^32^. To converge our differential cross sections phase shifts for partial waves from $\ell =0$ up to 3200 for the highest collision energy have been used. As one can see in Fig. 4a with its logarithmic scale along the vertical axis, the differential cross sections are largest for small $\theta _\mathrm{CM}$ of order a few degrees and have rapid oscillations for $\theta _\mathrm{CM}$ up to 20$^\circ$.

A complicating factor in the beam experiments by Han et al.^32^ is that small angle deflections of the heavy C_60_ do not lead to detectable loss of fullerenes from the supersonic beam. Han et al., account for this limitation by introducing a dimensionless device-dependent angular resolution function $\eta (\theta _\mathrm{CM})$ with values between 0 and 1 as function of $\theta _\mathrm{CM}$. We show this function in Fig. 4b. Here, $\eta (\theta _\mathrm{CM})=0$ implies that 100% of the loss of C_60_ from the beam due to scattering into angle $\theta _\mathrm{CM}$ is detected. A value of 1 implies that none of the loss is detected. Clearly, for the apparatus of Han et al., no signal is detected for $\theta _\mathrm{CM}<10^\circ$ even though the differential cross section is largest for these angles.

We derived $\eta (\theta _\mathrm{CM})$ from digitizing the data in Fig. 3 of Ref.^32^ and using the definition of the reduced deflection in its caption. In fact, we use a distance of 12.5 cm between the (mean) scattering location of a C_60_ molecule and the detector, assume a detector aperture diameter of 0.347 cm, and realize that polar scattering angle $\Theta$ of a scattered C_60_ molecule relative to the beam direction in the laboratory frame and $\theta _\mathrm{CM}$ in the center-of-mass frame are related by^41^

4 $$\begin{aligned} \tan \Theta = \frac{\sin \theta _\mathrm{CM}}{\cos \theta _\mathrm{CM}+1/\xi }. \end{aligned}$$

Here, ${\xi = m_\mathrm{Ar}/m_{\textrm{C}_{60}}\ll 1}$ is the ratio of the masses of $^{40}$Ar and $^{12}$C_60_. For $\xi <1$, by inspection $\Theta \in [0,\arcsin \xi ]$ so that in the laboratory frame the angular deflections of the heavy fullerene are small. Moreover, $\theta _\mathrm{CM}\in [0,\theta _\mathrm{max}]$ with $\theta _\mathrm{max}$ slightly larger than $\pi /2$ and found from $\cos \theta _\mathrm{max}=-\xi$. (The detector aperture diameter was not specified in Ref.^32^. We assumed that the largest reduced deflection in Fig. 3 of Ref.^32^ corresponds to the largest allowed value for $\Theta$. A derived diameter of 0.347 cm is not unreasonable for beam detectors.)

We then simulate the experiment signal of Ref.^32^ by constructing the apparent cross section

5 $$\begin{aligned} \sigma _\mathrm{app}(E)=2\pi \int _0^{\theta _\mathrm{max}} \sin (\theta _\mathrm{CM}) \textrm{d}\theta _\mathrm{CM} \{1-\eta (\theta _\mathrm{CM})\}\, \frac{\textrm{d}\sigma }{\textrm{d}\Omega } \,, \end{aligned}$$

Products $\{1-\eta (\theta _\mathrm{CM})\}\, \textrm{d}\sigma /\textrm{d}\Omega$ as function of $\theta _\mathrm{CM}$ are shown in Fig. 4c for the four relevant relative velocities or collision energies. As expected from the behavior of $\eta (\theta _\mathrm{CM})$, the experimental apparatus does not count collision events below 10$^\circ$. Moreover, the rapid angular oscillations in $\textrm{d}\sigma /\textrm{d}\Omega$ are only relevant for the smallest collision energy of $hc\times 3102$ cm$^{-1}$, while the products for the three larger collision energies are identical on the scale of the figure. It is worth noting that large-angle scattering processes are governed by small-impact parameter collisions of noble-gas atoms from the repulsive wall of the potential.

In Table 1 we list values for the semi-classical elastic cross section $\sigma _\mathrm{SC}(E)$, the elastic cross section $\sigma (E)$ based on quantum scattering calculations, and the apparent cross section $\sigma _\mathrm{app}(E)$ computed from our differential cross sections and the angular resolution function of the apparatus of Ref.^32^ at four large collision energies. Our standard uncertainty for $\sigma _\mathrm{app}(E)$ is due to the uncertainty in obtaining the resolution function. We first note that $\sigma _\mathrm{app}(E) \ll \sigma (E), \sigma _\mathrm{SC}(E)$ as expected from the data in Fig. 4.

Next we compare the theoretical apparent cross sections to the corresponding experimental data of Ref.^32^. The apparent cross sections from the experiment are larger than our computed values although the agreement improves for the largest collision energies. Both observations might be consistent with an underestimate of the uncertainty deriving the resolution function and specifically our estimate of the diameter of the detector aperture, but also with the absence of inelastic processes from transitions between ro-vibrational states of C_60_ induced by collisions with Ar in the theoretical simulations. These transitions are due to potential strengths $V_{l,m}(R)$ with $l,m\ne 0,0$. For the higher collision energies inelastic loss will be smaller. An underestimate of the uncertainty of the aperture diameter is more likely. Finally, the effects associated with the thermal distributions of velocities of both C_60_ and Ar are reported to be small, amounting to an uncertainty of 2 % in the measured apparent cross sections.

**Table 1:** Elastic cross sections and apparent cross sections as defined in the text at four collision energies for $^{12}$C_60_-$^{40}$Ar collisions in units of Å$^2$ obtained from the semiclassical (SC) formula in Eq. (2), our quantum-mechanical (QM) scattering calculations, and experimental (EXP) results of Han et al.^32^.

Method	*E*/*hc* (cm$^{-1}$) $\rightarrow$	3102	6330	6979	9115
	Observable $\downarrow$				
SC	$\sigma _\mathrm{SC}$	984	853	837	793
QM	$\sigma$	1160	1061	1019	953
QM	$\sigma _\mathrm{app}$	69 (3)	62 (3)	62 (3)	60 (3)
EXP	$\sigma _\mathrm{app}$	111 (3)	94 (2)	85 (3)	70 (4)

Numbers in parenthesis represent standard uncertainties in the data. The uncertainties in our computed $\sigma _\mathrm{app}$ represent the 5 % standard uncertainty in the angular resolution function $\eta (\theta _\mathrm{CM})$.

## Conclusion

We have performed quantum scattering calculations to describe elastic collisions between the rigid $^{12}$C_60_ fullerene and $^4$He and $^{40}$Ar noble-gas atoms. Such collisions are of interest in buffer gas cooling of fullerenes. In order to perform the quantum scattering calculations we determined the potential energy surfaces by density functional theory connected to the long-range dispersion potential based on van der Waals $C_6$ dispersion coefficients computed from frequency-dependent dynamic polarizabilities of the colliding particles. We realized that the anisotropic components of these potentials are small and can be omitted in the calculation of scattering observables. We determined differential cross sections, total elastic cross sections, and rate coefficients for both systems and compared those to values obtained from a semiclassical formula that only depends the reduced mass of the system and the $C_6$ dispersion coefficient. The semiclassical data underestimates thermalized rate coefficients by about 20%. The size of the rate coefficients is sufficiently large that buffer gas cooling will be efficient.

For C_60_ collisions with Ar we could compare total elastic cross sections computed from integrating the differential cross sections with the window apparatus functions to the experimental results at four collision energies performed in 1995 by Ke-Li Han et al.^32^ and we found fairly good agreement.

## Supplementary Information

Supplementary Information.

## Data Availability

Correspondence and requests for materials should be addressed to S.K.
