# Supplementary material for: Quantum scattering of icosahedron fullerene C60 with noble-gas atoms
Source: Sci Rep. 2024 Apr 23;14:9267. doi: 10.1038/s41598-024-59481-x (PMC11551206; doi:10.1038/s41598-024-59481-x)
Supplement: Supplementary file 1 — Supplementary Information. [file 41598_2024_59481_MOESM1_ESM.pdf]

Supplemental Material: Quantum Scattering of Icosahedron Fullerene C_{60} with Noble-Gas Atoms

Jacek Kłos,[1] Eite Tiesinga,[2,3] and Svetlana Kotochigova[1,*]

[1] *Department of Physics, Temple University, Philadelphia, Pennsylvania 19122, USA*

[2] *National Institute of Standards and Technology, Gaithersburg, Maryland 20899, USA*

[3] *Joint Quantum Institute, College Park, Maryland 20742, USA*

Potential energy surface

In Fig. 2 of this article we have shown one- and two-dimensional cuts through the three-dimensional ground-state potential energy surface $U(\vec{x})$ of the C_{60}-Ar and C_{60}-He systems. Here, we give details regarding the computation of these surfaces. The fullerene is rigid with the carbon atoms located at the equilibrium icosahedral isomer geometry of an isolated fullerene molecule so that $\vec{x} = (R, \theta, \phi)$ in spherical coordinates locates the noble-gas atom relative to the center of mass of C_{60}.

The potential energy surfaces (PESs) for both van-der-Waals complexes have been obtained with counterpoise-corrected supermolecular density functional theory (DFT) using the hybrid wB97XD [1] functional and 6-31G(d,p) basis set with the Gaussian 09 program [2, 3]. We started from the initial carbon geometry found in the Yoshida Library of fullerenes [4–6]. This initial structure served as a starting point for geometry optimization and frequency calculations. The counterpoise procedure corrects for the basis set superposition error.

The potentials have been calculated on an uniform grid of R from 5 Å to 35 Å with a step of 0.2 Å. Using the icosahedral I_h symmetry of C_{60} and our choice of the z axis in Fig. 1 the grid of azimuthal angles ϕ could be restricted to 0°, 9°, and 18° degrees. The polar angle θ was restricted to 0.00°, 10.00°, 20.41°, 31.72°, 35.00°, 43.02°, 50.00°, 63.44°, 80.00°, 90.00°, 100.81°, 121.72°, 142.63°, and 163.00° degrees. These angles correspond to either a center of the hexagon or pentagon rings, half the distance between a C-C bond or a C-vertex.

The PESs are expanded in terms of Racah normalized spherical harmonics $C_{lm}(\theta, \phi)$ conforming to the I_h group as defined in Eq. (1) of this article. Due to our choice of the z axis the non-vanishing expansion coefficients or strengths $V_{l,m}(R)$ occur at $l = 0, 6, 10, 12, 16, 18, 20, 22, \ldots$ with $m = n \times 5$,

*Electronic address: skotoch@temple.edu

where $n = 0, 1, 2, \ldots$. We expand our potentials with terms up to $l = 20$ and $m = 20$ and at each R have performed a least squares fit based on $U(R, \theta, \phi)$ found at the $3 \times 14 = 42$ angular data points. The long-range dispersion tails of the $V_{lm}(R)$ have been obtained from the dynamic polarizabilities of C_{60} and of the two noble-gas atoms.

The short-range behaviors of the non-zero radial expansion $V_{l,m}(R)$ coefficients have previously been discussed in Ref. [7]. The isotropic $V_{0,0}(R)$ coefficient, shown in Fig. 2 for both C_{60}-Ar and C_{60}-He, dominate near the minimum of the PES. The largest anisotropic contribution originates from $V_{10,0}(R)$.

---

[1] J.-D. Chai and M. Head-Gordon, Phys. Chem. Chem. Phys. **10**, 6615 (2008), URL http://dx.doi.org/10.1039/B810189B.

[2] M. J. Frisch, G. W. Trucks, H. B. Schlegel, G. E. Scuseria, M. A. Robb, J. R. Cheeseman, G. Scalmani, V. Barone, B. Mennucci, G. A. Petersson, et al., *Gaussian 09, Revision E.01* (2013), Gaussian, Inc., Wallingford CT, URL https://gaussian.com.

[3] Any mention of commercial products is for information only; it does not imply recommendation or endorsement by the National Institute of Standards and Technology.

[4] P. W. Fowler and D. E. Manolopoulos, *An atlas of fullerenes* (Courier Corporation, 2007).

[5] P. Schwerdtfeger, L. Wirz, and J. Avery, J. Comp. Chem. **34**, 1508 (2013), A website can be found at https://ctcp.massey.ac.nz/index.php?group=&page=fullerenes&menu=fullerenes, URL https://onlinelibrary.wiley.com/doi/abs/10.1002/jcc.23278.

[6] P. Schwerdtfeger, L. N. Wirz, and J. Avery, WIREs Computational Molecular Science **5**, 96 (2015), URL https://doi.org/10.1002/wcms.1207.

[7] L. R. Liu, P. B. Changala, M. L. Weichman, Q. Liang, J. Toscano, J. Kłos, S. Kotochigova, D. J. Nesbitt, and J. Ye, PRX Quantum **3**, 030332 (2022), URL https://link.aps.org/doi/10.1103/PRXQuantum.3.030332.
